# Topical *Trichosanthis Radix* Water Extract Attenuates Atopic Dermatitis‐Like Skin Inflammation: Marker Standardization, Network Pharmacology, and Preclinical Validation

**DOI:** 10.1155/mi/8744885

**Published:** 2026-07-09

**Authors:** Hye-Min Kim, Geun-Lip Kim, Tae-Young Gil, Ha-Yeon Sim, Hwan Lee, Dong-Sung Lee, Kyou-Young Lee, Hyo-Jin An

**Affiliations:** ^1^ College of Pharmacy and Institute of Integrated Pharmaceutical Sciences, Kyung Hee University, 26 Kyungheedae-ro, Dongdaemun-gu, Seoul, 02447, Republic of Korea, khu.ac.kr; ^2^ Department of Herbology, College of Korean Medicine, Sangji University, 83 Sangjidae-gil, Wonju-si, 26339, Republic of Korea, sangji.ac.kr; ^3^ Department of Ophthalmology and Otolaryngology and Dermatology, College of Korean Medicine, Sangji University, 83 Sangjidae-gil, Wonju-si, 26339, Republic of Korea, sangji.ac.kr; ^4^ Department of Biomedical and Pharmaceutical Sciences, College of Pharmacy, Kyung Hee University, Seoul, 02447, Republic of Korea, khu.ac.kr; ^5^ Department of Pharmacy, College of Pharmacy, Chosun University, Dong-gu, Gwangju, 61452, Republic of Korea, chosun.ac.kr

**Keywords:** atopic dermatitis, inflammation, JAK2–STAT1 signaling, network pharmacology, T*richosanthis Radix*

## Abstract

*Trichosanthis Radix* (TR) is a pharmacopeial crude drug traditionally used to clear heat, reduce swelling, expel pus, and generate fluids, suggesting potential relevance to inflammatory skin disorders such as atopic dermatitis (AD). In this study, we evaluated the AD‐related activity of a TR water extract using an integrated workflow combining network‐based target analysis, marker compound analysis, and experimental validation in vivo and in vitro. The overlap between predicted TR targets and AD‐associated genes was analyzed using a protein–protein interaction network, and extract was analyzed by high performance liquid chromatography (HPLC)–evaporative light scattering detector (ELSD). Biological effects were examined in DNCB‐induced murine dermatitis and TNF‐α/IFN‐γ‐stimulated HaCaT keratinocytes. Twenty‐five overlapping genes formed a compact interaction module enriched for immune signaling and lipid/nuclear receptor‐related pathways, and HPLC–ELSD detected and quantified L‐citrulline as a marker compound for TR extract standardization. Topical TR reduced dermatitis severity, attenuated epidermal hyperplasia, and decreased dermal mast cell accumulation, with more consistent effects at the higher dose. TR also lowered lesional chemokine transcripts, particularly CCL17/TARC and CCL5/RANTES, and was associated with reduced NLRP3 and IL‐1β. In addition, TR suppressed DNCB‐induced phosphorylation of MEK/ERK and JAK2–STAT1 and decreased p‐STAT1 immunofluorescence. In HaCaT cells, TR attenuated cytokine‐induced inflammatory effectors and phosphorylation of MEK/ERK and STAT1. These findings indicate that TR water extract mitigates AD‐relevant inflammatory signaling while improving a barrier‐associated outcome, supporting further constituent‐resolved and mechanism‐directed studies.

## 1. Introduction

Atopic dermatitis (AD) is a chronic, relapsing eczematous disorder in which pruritus is disease‐defining, and rapid control of cutaneous inflammation and itch remains a core therapeutic objective [1]. The population‐level impact of AD is substantial: recent global burden estimates indicate that the absolute number of individuals living with AD increased from 1990 to 2021, and epidemiologic syntheses continue to document wide geographic variability in prevalence and incidence across children and adults [2, 3]. Beyond prevalence, AD imposes a meaningful public health burden through increased direct and indirect costs and marked impairment in the quality of life [4]. Contemporary frameworks conceptualize AD as a heterogeneous inflammatory disease with substantial phenotypic variation [5] and emphasize epidermal barrier dysfunction alongside immune dysregulation as key, interrelated features of disease pathogenesis [6, 7]. Accordingly, guidelines prioritize topical anti‐inflammatory therapy as foundational management, while systemic options, including biologics and oral Janus kinase inhibitors, have expanded for moderate to severe or refractory disease, accompanied by the need for individualized risk–benefit assessment and monitoring [1, 8, 9].

In parallel with immune‐centered paradigms, environmental and symptom‐amplifying factors remain clinically significant, particularly in relation to itch. Heat and sweating are widely discussed as exacerbating factors for itch in AD, and broader climate‐related exposures have been implicated as potential contributors to epithelial barrier disruption and allergic disease flares in respiratory and dermatologic contexts [10, 11]. In general inflammatory biology, “heat” is also classically recognized among the cardinal features of acute inflammation, alongside redness, swelling, pain, and loss of function [12]. Within traditional East Asian medicine, “heat” is similarly treated as a clinically relevant dimension of inflammatory symptomatology, and Trichosanthis Radix (TR) (Tian Hua Fen; the dried root of *Trichosanthes kirilowii*) has been described in pharmacopeial and review sources as having traditional functions that include “clearing heat,” reducing swelling, and expelling pus [13]. Therefore, traditional descriptions of heat would be included as a point of view—amplified inflammatory signaling and barrier stress—which can be indicated for evaluating TR within a multitarget, systems‐level framework.

Alongside targeted synthetic and biologic modalities, natural product‐derived candidates remain an active area of investigation in AD, with contemporary reviews cataloging multiple compounds and botanical sources evaluated in experimental systems [14]. For TR specifically, prior experimental literature provides a rationale for further evaluation: saponin‐rich fractions from TR have demonstrated antioxidant activity in standard in vitro assays and showed in vivo modulation of oxidative stress‐related readouts in a CCl_4_‐induced oxidative stress model [15]. Trichosanthin, a protein component derived from *Trichosanthes* species, has been reported to alter macrophage cytokine/chemokine expression patterns under stimulation, characterized by increased IL‐10 and MCP‐1 with decreased IL‐12 and TNF‐α, and has also been reported to prevent or delay acute rejection in an MHC‐mismatched mouse skin allograft model [16, 17]. In addition, an herb‐pair study incorporating TR reported anti‐inflammatory activity in a zebrafish inflammation model; however, the evidence in this context derives from a combination rather than TR alone [18].

On this basis, we evaluated TR water extract directly in AD‐like dermatitis models, integrating systems‐level target/pathway analyses with in vivo efficacy testing in a DNCB‐induced dermatitis model and complementary mechanistic validation in cytokine‐stimulated keratinocytes. In the in vivo model, we assessed clinical severity and barrier‐associated readouts alongside histopathology and lesional inflammatory markers, including chemokine transcripts and protein‐level inflammatory signaling outputs. In parallel, we used TNF‐α/IFN‐γ–stimulated HaCaT keratinocytes to probe TR‐associated modulation of cytokine‐driven inflammatory programs and pathway activation, aligning in vitro signaling readouts with the in vivo pathway‐focused findings.

## 2. Materials and Methods

### 2.1. Identification of TR‐Related Targets and AD‐Associated Genes

Putative targets associated with TR were identified through a compound‐based network pharmacology workflow. Candidate TR compounds were retrieved from the Traditional Chinese Medicine Systems Pharmacology database (TCMSP) (accessed July 2025) and screened using standard ADME‐based criteria, namely, oral bioavailability (OB) ≥ 30% and drug‐likeness (DL) ≥ 0.18. Based on these thresholds, two ADME‐filtered candidate compounds of TR, spinasterol and schottenol, were retained (Supporting Information Table S1). The putative protein targets of these compounds were predicted using SwissTargetPrediction, with *Homo sapiens* as the target organism. After the removal of duplicate entries, 61 TR‐related putative targets were obtained (Supporting Information Table S2). In parallel, AD‐associated genes were retrieved from the GeneCards database (accessed July 2025) using the search term “atopic dermatitis.” Genes with a relevance score ≥ 0.278911412 were retained, yielding 1547 AD‐associated genes (Supporting Information Table S3). The overlap between TR‐related putative targets and AD‐associated genes was identified and visualized using Venny 2.1, resulting in 25 overlapping genes that were considered putative TR–AD‐associated targets for downstream network analysis (Supporting Information Table S4). Protein–protein interaction data for these overlapping targets were retrieved from the STRING database, with *Homo sapiens* selected as the target organism and the minimum required interaction score set to ≥ 0.4. The PPI network was imported into Cytoscape version 3.10.4 for topological analysis. To identify hub genes within the network, the cytoHubba plugin was used with three topological parameters: degree, betweenness centrality, and closeness centrality. The top 10 genes ranked by each algorithm were extracted and are provided in Supporting Information Table S5.

### 2.2. Functional Enrichment and Pathway Analysis

Gene Ontology (GO) and Kyoto Encyclopedia of Genes and Genomes (KEGG) pathway enrichment analyses were performed using the DAVID bioinformatics resource, with *Homo sapiens* selected as the species. GO terms and KEGG pathways with a nominal *p*‐value < 0.05 were retained for exploratory enrichment interpretation. For GO analysis, the top 10 terms in each category—biological process (BP), cellular component (CC), and molecular function (MF)—were selected based on enrichment score, defined as −log_10_ nominal *p*‐value (Supporting Information Table S6). For KEGG analysis, the top nine pathways were selected based on the nominal *p*‐value (Supporting Information Table S7). Adjusted values, including Bonferroni, Benjamini–Hochberg, and FDR values, were provided alongside nominal *p*‐values in the supplementary tables for transparent reporting; however, the primary ranking and exploratory interpretation were based on the nominal *p*‐values.

### 2.3. Preparation of TR Water Extract

Dehydrated roots of *Trichosanthes kirilowii* Maximowicz were purchased from Gyeonggiherb (Jeju Island, Republic of Korea). The raw material was extracted in water at 100°C for 4 h, filtered using a Whatman filter paper, and lyophilized (COOL ACE CA‐1500, EYELA, Tokyo, Japan). TR was prepared as a water extract to reflect its traditional decoction‐based use and pharmacopeial relevance while avoiding residual organic solvents in the extract intended for topical preclinical evaluation. Thus, this extraction method was selected to support both experimental reproducibility and translational applicability [13]. The extraction yield (crude material to dried powder) was 9.45% (w/w). The lyophilized product was designated as TR water extract. For in vivo experiments, TR was administered as a low‐dose preparation (TRL, 100 mg/kg) or a high‐dose preparation (TRH, 200 mg/kg). TR was diluted in sterile saline before topical application and applied at a fixed volume per application, as specified below.

### 2.4. Analysis Using High Performance Liquid Chromatography (HPLC)–Evaporative Light Scattering Detectors (ELSDs)

HPLC–ELSD analysis was performed to confirm the presence of L‐citrulline and determine its content as a marker compound in the TR extract. The TR extract was dissolved in distilled water to a concentration of 10,000 ppm, and L‐citrulline standard solutions were prepared in distilled water over a concentration range of 15.625–1000 ppm. The analysis was carried out using a Waters 2996 HPLC system coupled to a Waters 2424 ELSD detector (Waters Corp., Milford, MA, USA). Separation was performed on a Kinetex 5 µm C18 100 Å column (250 mm× 4.6 mm I.D., Phenomenex Inc., Torrance, CA, USA) using an isocratic mobile phase of distilled water containing 0.3% trifluoroacetic acid at a flow rate of 0.7 mL/min for 20 min. The injection volume was 10 µL. The ELSD was operated with a drift tube temperature of 100°C and a nitrogen gas pressure of 50 psi. To evaluate the specificity of the L‐citrulline peak in the TR extract, chromatograms of the blank, L‐citrulline standard, TR extract, and L‐citrulline‐spiked TR extract were compared. Spike‐in tests were performed by adding known amounts of L‐citrulline standard to the TR extract at three levels, corresponding to 50, 100, and 200 µg. The spiked samples were analyzed under the same HPLC–ELSD conditions. The specificity of the L‐citrulline peak was assessed based on the consistent retention time, the increase in the peak area after standard addition, and the absence of interfering peaks around the L‐citrulline retention time.

### 2.5. Limit of Quantification (LOQ) and Limit of Detection (LOD)

For calibration purposes, L‐citrulline standard solutions were analyzed at concentrations ranging from 15.625 to 1000 ppm. Considering the nonlinear response characteristics of ELSD, the calibration curve was constructed using a log–log regression model by plotting the logarithm of the peak area against the logarithm of the corresponding L‐citrulline concentration. The calibration equation was expressed as log *y* = 1.4385 log *x* + 2.1173, where *x* is the L‐citrulline concentration and *y* is the ELSD peak area. Good linearity was obtained within the tested range with an *R* ^2^ value of 0.9990. The LOD and LOQ were calculated based on the calibration curve as 3.3 × (standard deviation/slope) and 10 × (standard deviation/slope), respectively. The calculated log concentration values were back‐transformed and expressed as ppm.

### 2.6. Content Evaluation and Recovery Test Using HPLC–ELSD Method

The L‐citrulline content in the TR water extract was evaluated using the peak area values obtained from HPLC–ELSD analysis. The TR extract was analyzed five times at a concentration of 10,000 ppm. The L‐citrulline concentration in the sample solution was calculated using the log–log calibration equation established with L‐citrulline standard solutions. The content of L‐citrulline in the TR extract was calculated as follows: content (mg/g extract) = calculated L‐citrulline concentration (µg/mL)/extract concentration (mg/mL). The accuracy of the method was evaluated by a spike‐in recovery test using the standard addition method. Known amounts of L‐citrulline standard, corresponding to 50, 100, and 200 µg, were added to the TR extract and analyzed under the same HPLC–ELSD conditions. The recovery was calculated as [(amount found in the spiked sample − amount found in the unspiked sample) / amount added] × 100. The accuracy and reproducibility of the method were assessed based on the recovery and relative standard deviation (RSD) values.

### 2.7. DNCB‐Induced AD‐Like Mouse Model and Topical Treatment

Male BALB/c mice (6 weeks old; 17–22 g) were obtained from SAMTAKO (Gyeonggi‐do, Republic of Korea) and maintained under controlled conditions (20–25°C, 40%–60% humidity, and 12 h light/dark cycle). All animal procedures were approved by the Institutional Animal Care and Use Committee (KHMC‐IACUC‐2023‐027). Mice were randomly allocated to five groups (*n* = 7 per group): normal, DNCB, DNCB + dexamethasone (Dexa), DNCB + TRL, and DNCB + TRH. Dexa served as a positive control. To induce AD‐like dermatitis, the dorsal skin was shaved and sensitized with DNCB following a previously reported protocol. Briefly, 100 μL of 1.0% DNCB was topically applied twice weekly for 1 week, followed by a challenge with 100 μL of 0.5% DNCB three times weekly for an additional 3 weeks. For topical treatment, TR and Dexa were applied five times per week during the treatment period. On the days of 0.5% DNCB challenge, TR or Dexa was applied to the same shaved dorsal lesion area 4 h after DNCB application. On non‐DNCB treatment days, TR or Dexa was applied once daily to the same dorsal area. The mouse body weight was measured once weekly and used to calculate the dose for that week. TR and Dexa formulations were freshly prepared each day in sterile saline according to the weekly body weight‐based dose calculation. TRL and TRH were applied at nominal doses of 100 and 200 mg/kg, respectively, and Dexa was applied at 5 mg/kg as a positive control based on previous studies using Dexa in comparable DNCB‐induced AD mouse models. Each formulation was applied in a fixed volume of 100 μL per mouse. The treated dorsal area was approximately 8 cm^2^, corresponding to approximately 2 cm × 4 cm. Based on this treated area and a representative 20 g mouse, these doses corresponded to approximately 2 mg/site and 0.25 mg/cm^2^ for TRL, 4 mg/site and 0.50 mg/cm^2^ for TRH, and 0.1 mg/site and 0.0125 mg/cm^2^ for Dexa, with minor variations according to weekly body weight. The vehicle control group received 100 μL of sterile saline. The treated skin was not occluded after application. The formulations were prepared without additional organic solvent or pH‐modifying excipients, and the pH was not separately measured or adjusted. At the end of week 4, mice were euthanized by cervical dislocation, and blood and dorsal skin tissues were collected for downstream analyses.

### 2.8. Assessment of Clinical Severity, Pruritus‐Related Behavior, and Skin Barrier–Associated Readouts

Clinical dermatitis severity was evaluated weekly using a SCORAD‐based scoring system by grading erythema, edema/papulation, excoriation, lichenification, oozing/crust formation, and dryness on a 0–3 scale (0, none; 1, mild, <20%; 2, moderate, 20%–60%; and 3, severe, >60%). For each mouse, the six component scores were averaged to obtain the mean clinical dermatitis score rather than summed as a total score. Clinical scoring was independently performed by three investigators blinded to the experimental groups, and the mean value of the three assessments was used for the analysis. To assess pruritus‐related behavior, mice were acclimated in transparent cages for at least 10 min after the 4‐week treatment period, and scratching of the dorsal area with the hind paws was video‐recorded for 10 min after completion of the treatment period; scratching frequency was then quantified. A scratching bout was defined as a hind‐paw scratching movement directed toward the dorsal lesion area, and repeated scratching movements were counted as a single bout until the paw was placed back on the cage floor or the animal resumed normal activity. Video‐based scratching analysis was independently performed by three investigators blinded to the experimental groups, and the mean value of the three counts was used for the analysis. In addition, transepidermal water loss (TEWL), rather than a clinical skin score surrogate, was measured at the end of week 4 using GPskin Barrier Light (Gpskin, Seoul, Republic of Korea) by placing the probe at the center of the shaved dorsal region. Before the measurement, mice were acclimated under controlled room conditions, and measurements were performed at the same dorsal site to minimize site‐dependent variability. TEWL values were used as an index of epidermal permeability barrier dysfunction and were first recorded as instrument‐derived TEWL values and then processed for graphical presentation as fold change relative to the normal control group.

### 2.9. Histological Evaluation of Skin Lesions

Dorsal skin tissues were harvested after euthanasia, fixed in 10% neutral buffered formalin, embedded in paraffin, and sectioned. Sections were stained with hematoxylin and eosin (H&E) to evaluate histopathological alterations and with toluidine blue to assess mast cell infiltration. Mast cells were quantified as the cell density (cells/mm^2^) from averaged counts per sample. Images were acquired using an EVOS M5000 optical microscope (Thermo Fisher Scientific) under identical magnification settings across groups.

### 2.10. Reverse Transcription‐Quantitative PCR (RT‐qPCR)

Total RNA was extracted from dorsal skin tissues and HaCaT cells using the Easy Blue kit (Intron Biotechnology, Inc., Seoul, Republic of Korea) and quantified on an Epoch microvolume spectrophotometer (BioTek Instruments, Inc., Winooski, VT, USA). Following genomic DNA removal, 2 μg of total RNA was reverse‐transcribed with a d(T)16 primer and avian myeloblastosis virus reverse transcriptase to obtain cDNA. Quantitative PCR was conducted on a 7500 Real‐Time PCR System (Applied Biosystems; Thermo Fisher Scientific, Inc., Waltham, MA, USA) using SYBR Premix Ex Taq. Relative expression of TARC/CCL17, MDC/CCL22, and RANTES/CCL5 in dorsal skin tissues and TNF‐α/IFN‐γ‐stimulated HaCaT keratinocytes was calculated using the ΔΔCq method and normalized to GAPDH using ABI Gene Express 2.0 software. Primer information is provided in Supporting Information Table S8, and the specificity of amplification was confirmed by melt curve analysis.

### 2.11. Western Blot Analysis

Dorsal skin tissues were lysed in PRO‐PREP protein extraction solution (Intron Biotechnology, Inc., Seoul, Republic of Korea) and incubated for 20 min at 4°C. For phosphorylation analyses, lysis and processing were conducted under cold conditions and performed promptly after homogenization. Lysates were clarified by centrifugation (11,000 × *g*, 30 min, 4°C), and the supernatants were rapidly frozen. Protein concentrations were determined using a Bio‐Rad protein assay reagent (Bio‐Rad Laboratories, Inc.) according to the manufacturer’s instructions. Proteins were separated by 10%–12% SDS‐PAGE and transferred to PVDF membranes. Membranes were blocked with 2.5%–5.0% skim milk for 30 min at room temperature and incubated with primary antibodies (1:1000–1:2500) overnight at 4°C. After washing with Tween 20/Tris‐buffered saline, membranes were incubated with HRP‐conjugated secondary antibodies (1:2000–1:5000) for 2 h at room temperature. Signals were detected using enhanced chemiluminescence (GE Healthcare Life Sciences, Chalfont, UK). For western blot analysis of mouse dorsal skin tissues, each lane represents the protein lysate from an individual mouse and not a pooled sample. The animal experiments included 5–7 mice per group, and western blotting was performed using selected individual skin lysates that met the protein quantity and quality requirements for western blot analysis. The blots shown in the figures are representative images of the analyzed biological samples. For cell‐based western blotting, each lane represents an independent culture replicate, and experiments were performed using three independent biological replicates, unless otherwise stated. Band intensities were quantified by densitometry using ImageJ. For each target band, the integrated density was measured after local background subtraction. Total protein signals were normalized to β‐actin. For phosphorylated signaling proteins, phospho‐protein signals were normalized to their corresponding total protein levels, and the corresponding total protein signals were normalized to β‐actin. The normalized values were then expressed relative to the normal control group, disease‐control group, or cytokine‐stimulated control group, as indicated in the relevant figure panels. Full‐length uncropped western blot images, including molecular weight information, are provided in the Supporting Information.

### 2.12. Immunofluorescence Detection in Lesional Skin

Paraffin‐embedded dorsal skin sections were deparaffinized, rehydrated through graded alcohol, and subjected to heat‐induced antigen retrieval using standard conditions suitable for phospho‐STAT1 staining. Sections were blocked and incubated with an antibody against phospho‐STAT1 (Tyr701), followed by fluorophore‐conjugated secondary antibodies. Nuclei were counterstained with DAPI, and images were acquired using an EVOS M5000 imaging system under identical exposure settings across groups. p‐STAT1 immunoreactivity was evaluated qualitatively across treatment conditions.

### 2.13. *In Vitro* Experiment

HaCaT human keratinocytes (species: *Homo sapiens*; tissue of origin: epidermal keratinocytes; RRID: CVCL_0038) were kindly provided by Prof. Kyung‐Tae Lee (Kyung Hee University, Seoul, Korea). HaCaT cells were maintained in DMEM supplemented with 10% fetal bovine serum, penicillin (100 U/mL), and streptomycin (100 μg/mL) at 37°C in a humidified incubator with 5% CO_2_. HaCaT cell viability was evaluated using both MTT and CCK‐8 assays to exclude concentration‐dependent cytotoxicity under the same exposure conditions used for signaling analyses. Cells were seeded in 96‐well plates, allowed to adhere overnight, and treated with TR at 0–1000 μg/mL for 24 h, followed by MTT or CCK‐8 analysis according to standard colorimetric procedures. Based on these assays, TR showed no significant cytotoxicity at the concentrations used for subsequent TNF‐α/IFN‐γ‐stimulated signaling experiments, including 250, 500, and 1000 μg/mL. For cytokine‐driven stimulation, TR‐pretreated cells were costimulated with TNF‐α (10 ng/mL) and IFN‐γ (10 ng/mL) for the indicated durations, and lysates were collected for immunoblot analyses of inflammasome‐associated proteins and phosphorylation events.

### 2.14. Statistical Analysis

Results are presented as the mean ± SD. Statistical analyses were conducted using GraphPad Prism version 5.0 (GraphPad Software, San Diego, CA, USA). Longitudinal outcomes, including body weight and mean clinical dermatitis score, were analyzed using two‐way repeated‐measures ANOVA, followed by Dunnett’s multiple‐comparisons test. The dermatitis score was analyzed as a mean clinical score derived from six semi‐quantitative clinical item scores. Endpoint measurements were analyzed using one‐way ANOVA, followed by Dunnett’s post hoc test for comparisons with the relevant control group. For animal experiments, *n* represents the number of individual mice per group. For cell‐based experiments, *n* represents independent biological experiments, unless otherwise stated. Statistical significance was set at *p* < 0.05. For enrichment analyses, adjusted *p*‐values and/or FDR values were recorded when available from enrichment platforms.

## 3. Results

### 3.1. Systems‐Level Identification of Shared TR–AD Targets and Pathway Architecture

**Figure 1:**
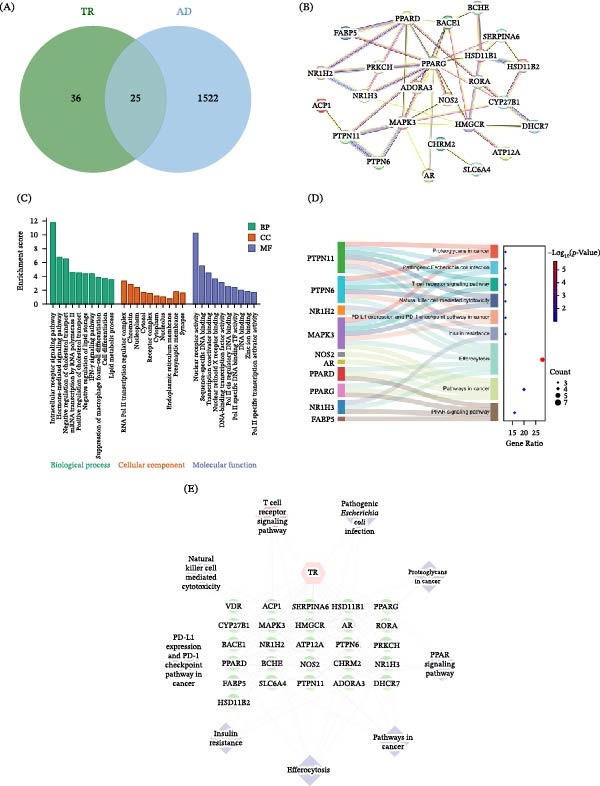
Identification and functional annotation of common targets linking TR to AD. (A) Venn diagram quantifying TR‐associated targets and AD‐associated genes and their overlap (*n* = 25), with overlapping gene symbols listed below. (B) PPI network for the overlapping genes generated in STRING to evaluate functional connectivity among candidates. (C) GO term enrichment across BP/CC/MF categories, highlighting top‐ranked terms by enrichment score. (D) KEGG pathway enrichment depicted using an alluvial representation of gene–pathway assignments and a dot plot summarizing gene ratio, gene counts, and statistical strength. (E) Composite TR–target–pathway network integrating overlapping targets with enriched KEGG pathways to provide a consolidated mechanistic view.

Using a common‐target filter, we identified 25 overlapping genes linking TR (the pharmacopeial crude drug name for the dried root of *Trichosanthes kirilowii* Maximowicz) in the target space to the AD gene space (Figure 1A). The interaction topology of these genes showed a compact, multiedge PPI structure (Figure 1B), indicating that the overlap set is enriched for functionally interacting proteins. Within this structure, transcriptional regulators and signaling mediators, particularly PPARG, PPARD, MAPK3, PTPN6/PTPN11, NR1H3, and NOS2, were positioned as central elements, implying potential coordination across signaling and transcriptional layers. GO enrichment highlighted immune activation and inflammatory regulation (Figure 1C). Terms such as the T cell receptor signaling pathway, IFN‐γ signaling pathway, and regulation of inflammatory response were among the top BPs, accompanied by lipid‐related processes, including regulation of cholesterol storage and lipid metabolic regulation. CC enrichment emphasized nuclear and nucleoplasmic localizations, transcriptional regulatory complexes, and receptor‐associated compartments, while MF terms were dominated by nuclear receptor activity, sequence‐specific DNA binding, and transcriptional coregulator interactions. Moreover, KEGG analysis consolidated these signals into a limited number of interpretable pathway groups (Figure 1D); lipid/nuclear receptor–centered pathways (PPAR signaling and insulin resistance), inflammatory resolution programs (efferocytosis), and immune signaling/effector pathways (T cell receptor signaling, natural killer cell‐mediated cytotoxicity, and checkpoint‐related signaling). The integrated TR–target–pathway network (Figure 1E) provided a systems‐level overview and generated the hypothesis that TR‐associated targets may intersect AD‐related inflammatory, immune, and barrier‐associated pathways. These computational predictions should be interpreted as hypothesis‐generating and require experimental validation.

### 3.2. HPLC–ELSD Profiling and Quantification of L‐Citrulline in TR

**Figure 2:**
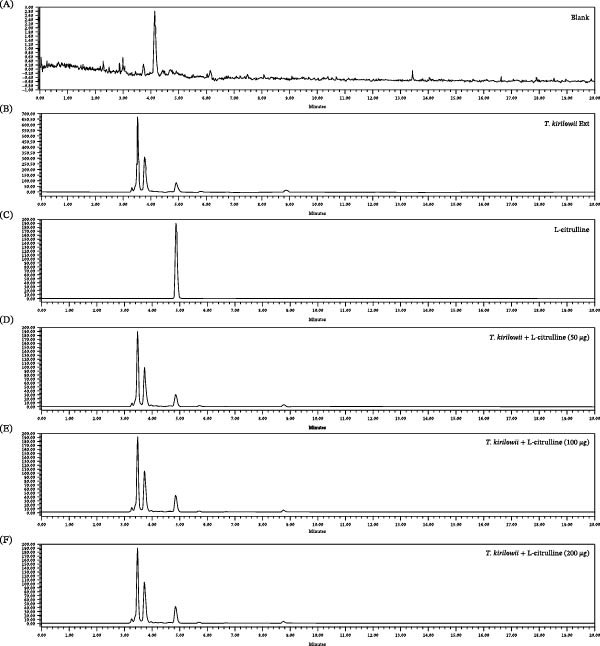
HPLC–ELSD chromatograms of L‐citrulline standard, *T. kirilowii* water extract, and L‐citrulline‐spiked *T. kirilowii* water extract. (A) Blank, (B) *T. kirilowii* water extract, (C) L‐citrulline standard, (D) *T. kirilowii* water extract spiked with 50 µg of L‐citrulline, (E) *T. kirilowii* water extract spiked with 100 µg of L‐citrulline, and (F) *T. kirilowii* water extract spiked with 200 µg of L‐citrulline. The L‐citrulline peak was detected at approximately 4.88 min, and the peak area increased after standard addition, supporting the assignment and specificity of the L‐citrulline peak in the extract.

HPLC–ELSD analysis was performed to detect and quantify L‐citrulline as a marker compound in the water extract of TR. In the ELSD chromatogram of TR, several peaks were detected, and the peak corresponding to L‐citrulline was observed at approximately 4.88 min, consistent with the retention time of the L‐citrulline standard (Figure 2). To further support the assignment and specificity of this peak, spike‐in analysis was performed by adding the L‐citrulline standard to the TR extract. The spiked samples showed an increase in the peak area at the same retention time without additional interfering peaks, supporting the assignment of the L‐citrulline peak in the TR extract.

For quantitative analysis, L‐citrulline standard solutions were analyzed over the concentration range of 15.625–1000 ppm. Considering the nonlinear response characteristics of ELSD, the calibration curve was constructed using a log–log regression model by plotting the logarithm of the peak area against the logarithm of the L‐citrulline concentration. The calibration equation was expressed as log *y* = 1.4385 log *x* + 2.1173, where *x* is the L‐citrulline concentration and *y* is the ELSD peak area. Good linearity was obtained within the tested range, with an *R* ^2^ value of 0.9990 (Table 1). The LOD and LOQ were calculated as 1.84 and 6.38 ppm, respectively, after back‐transformation from the log concentration values (Table 1).

**Table 1:** Calibration curves, limit of detection (LOD), and limit of quantification (LOQ) of L‐citrulline.

Analyte	Regression equation	*R* ^2^	Linear range (ppm)	LOD^a^	LOQ^b^
L‐citrulline	log *y* = 1.4385 log *x* + 2.1173	0.9990	15.625–1000	1.8437	6.3839

^a^LOD refers to the limits of detection, *S*/*N* = 3.3.

^b^LOQ refers to the limits of quantification, *S*/*N* = 10.

The L‐citrulline content in the TR water extract was calculated using the log–log calibration equation. As a result, the L‐citrulline content was determined to be 32.13 ± 0.07 mg/g extract, with an RSD of 0.21% (*n* = 5) (Table 2). In addition, the accuracy of the method was evaluated by spike‐in recovery tests at three levels, corresponding to 50, 100, and 200 µg of the L‐citrulline standard. The recovery values were calculated by comparing the measured amount in the spiked samples with that in the unspiked TR extract, and the results are presented in Table 3. The average recoveries were 90.46%, 95.46%, and 102.47% at the 50, 100, and 200 µg spike levels, respectively, with RSD values of 1.87%, 0.33%, and 0.51%. These recovery and RSD values supported the accuracy and reproducibility of the HPLC–ELSD method for L‐citrulline quantification in TR extracts.

**Table 2:** Content of L‐citrulline in water extract of *T. kirilowii* (*n* = 5).

Concentration of *T. kirilowii* extract (ppm)	Number	Contents (mg/g)	Average (mg/g)	Standard deviation	%RSD
10,000	1	32.1777	32.1282	0.0666	0.2072
	2	32.0987			
	3	32.2187			
	4	32.0791			
	5	32.0667			

**Table 3:** Recovery of L‐citrulline in water extract of *T. kirilowii* (*n* = 3).

Added amount of L‐citrulline (μg)	Number	Found amount	Recovery (%)	Average recovery (%)	Deviation	%RSD
50	1	45.7782	91.5564	90.4585	1.6959	1.8748
	2	45.6569	91.3138			
	3	44.2526	88.5052			
100	1	95.8079	95.8079	95.4584	0.3142	0.3291
	2	95.1995	95.1995			
	3	95.3677	95.3677			
200	1	206.1175	103.0587	102.4723	0.5216	0.5090
	2	204.5961	102.2981			
	3	204.1203	102.0601			

### 3.3. TR Attenuates DNCB‐Induced Clinical Dermatitis Severity and Improves Barrier‐Associated Readouts

**Figure 3:**
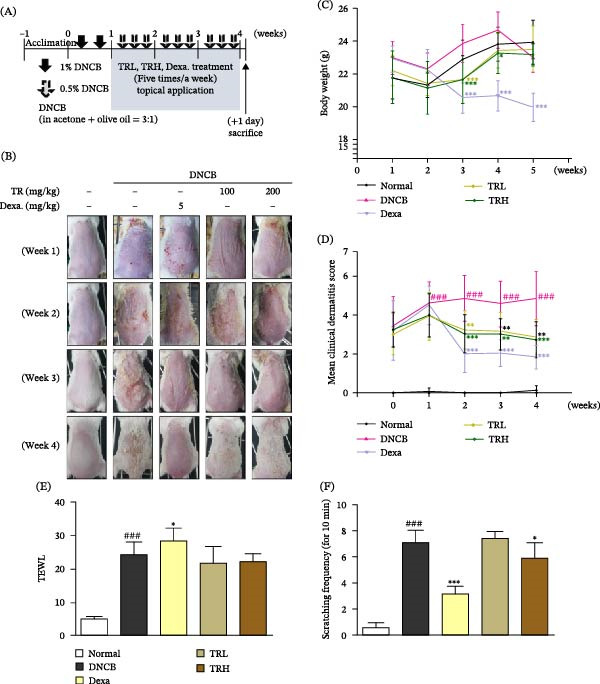
Effects of topical TR on clinical skin inflammation in DNCB‐challenged mice. (A) Timeline of DNCB sensitization and topical dosing regimen (administered five times per week), with terminal sampling 1 day after the last application. (B) Weekly gross images of dorsal lesions (weeks 1–4) across experimental groups (NOR, DNCB, Dexa, TRL; TR 100 mg/kg, TRH; TR 200 mg/kg). (C) Longitudinal body weight monitoring. (D) Longitudinal mean clinical dermatitis score, calculated by averaging six clinical item scores, each graded from 0 to 3. (E) Endpoint TEWL (g/m^2^/h) as an index of barrier dysfunction. (F) Quantification of pruritus‐related scratching behavior after the 4‐week treatment period. Data are presented as the mean ± SD. ###*p* < 0.001 versus the normal control group;  ^∗^ *p* < 0.05,  ^∗∗^ *p* < 0.01, and  ^∗∗∗^ *p* < 0.001 versus the DNCB‐treated group.

The workflow of induction and treatment is summarized in Figure 3A. DNCB application produced a robust dermatitis‐like phenotype, evident as progressive erythema, erosion/excoriation, and scaling in weekly photographs (Figure 3B). Compared with the DNCB control, topical TR at both doses reduced the visible extent and intensity of lesions over the course of treatment. Dexa improved gross inflammatory features but did not uniformly restore normal‐looking skin at each weekly assessment. Across the experimental period, body weight remained broadly stable in the normal and DNCB groups, while the Dexa group trended lower at later time points (Figure 3C). Dermatitis scores rose following DNCB challenge and stayed persistently high in the DNCB control group; both TR regimens decreased clinical scores during the treatment phase (Figure 3D). TEWL measurements at the endpoint confirmed substantial barrier disruption in DNCB‐challenged mice; TR groups showed a lower mean TEWL than DNCB, whereas Dexa remained associated with elevated TEWL in this dataset (Figure 3E). Because pruritus‐related scratching is a defining behavioral feature of AD‐like dermatitis, scratching bouts were quantified after the 4‐week treatment period. The DNCB challenge increased scratching behavior compared with the normal control group, confirming the development of pruritus‐associated behavioral symptoms in this model. Dexa and TRH significantly reduced scratching behavior compared with the DNCB control, suggesting partial improvement of pruritus‐related behavior (Figure 3F).

### 3.4. TR Mitigates DNCB‐Driven Epidermal Hyperplasia and Reduces Dermal Mast Cell Infiltration

**Figure 4:**
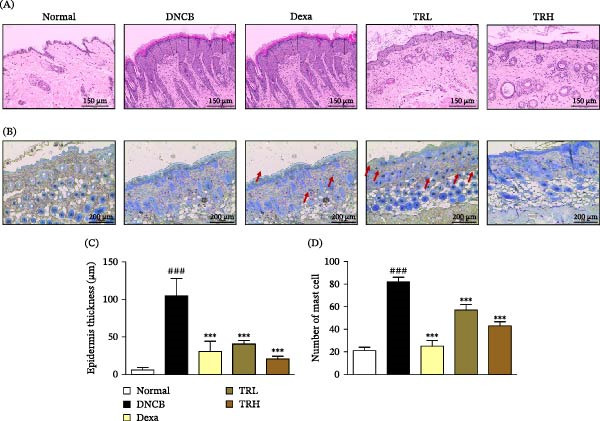
Effects of TR on histopathological inflammatory features in DNCB‐challenged mice. (A) Representative H&E‐stained sections of dorsal skin across experimental groups, highlighting epidermal architecture and hyperplasia (thickness demarcated by black lines). (B) Toluidine blue staining illustrating dermal mast cell distribution (arrows). (C) Group comparison of epidermal thickness. (D) Group comparison of mast cell abundance. Scale bars: 150 µm in H&E‐stained images and 200 µm in toluidine blue‐stained images. Data are presented as the mean ± SD. ###*p* < 0.001 versus the normal control group;  ^∗∗∗^ *p* < 0.001 versus the DNCB‐treated group.

Histological assessment revealed pronounced epidermal remodeling after DNCB exposure. In H&E‐stained sections, the DNCB group displayed marked epidermal hyperplasia with an expanded stratified layer compared with that of normal skin (Figure 4A), which was reflected by a substantial increase in quantified epidermal thickness (Figure 4C). Dexa reduced this hyperplastic response toward near‐baseline levels. TR treatment also decreased epidermal thickness relative to DNCB, with TRH producing a larger reduction than that of TRL, indicating a dose‐responsive trend in the epidermal compartment. Toluidine blue staining further indicated that DNCB challenge was accompanied by increased dermal mast cell presence (Figure 4B), consistent with enhanced inflammatory cell recruitment and retention in the lesional skin. Quantitatively, mast cell numbers were elevated in the DNCB group versus normal (Figure 4D). Dexa lowered mast cell counts to values close to the normal range. Both TR regimens reduced mast cell abundance compared with DNCB, with TRH again showing a greater reduction than TRL; however, mast cell counts in TR‐treated groups remained above the normal baseline in this dataset.

### 3.5. TR Attenuates Lesional Chemokine Transcript Expression and Suppresses Inflammasome‐Associated Protein Induction in DNCB‐Challenged Tissue

**Figure 5:**
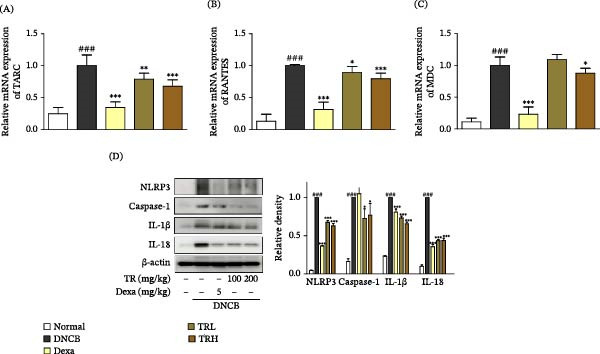
Effects of TR on lesional chemokine transcript expression and inflammasome‐associated protein induction in DNCB‐challenged mice. (A–C) Relative mRNA expression of TARC (CCL17), RANTES (CCL5), and MDC (CCL22) in endpoint dorsal skin quantified by qRT‐PCR, normalized to a housekeeping gene and expressed relative to the normal group. (D) Representative western blots of NLRP3, caspase‐1, IL‐1β, and IL‐18 in dorsal skin lysates from each group; β‐actin was used as a loading control. Densitometric quantification of each target protein normalized to β‐actin. Densitometry was performed using ImageJ. Data are presented as mean ± SD. ###*p* < 0.001 versus the normal control group;  ^∗^ *p* < 0.05,  ^∗∗^ *p* < 0.01, and  ^∗∗∗^ *p* < 0.001 versus the DNCB‐treated group.

DNCB challenge robustly upregulated AD‐associated chemokine transcripts in dorsal skin, with TARC (CCL17), RANTES (CCL5), and MDC (CCL22) mRNA levels increased relative to normal controls (Figure 5A–C). Dexa produced the most consistent suppression across all three transcripts compared with the DNCB group. TR treatment was associated with reduced chemokine expression in the lesional skin, showing a clearer reduction for TARC and RANTES and a comparatively more variable pattern for MDC, where the TRH group exhibited a more apparent decrease than the TRL group. These findings indicate that TR partially attenuated the transcriptional upregulation of AD‐associated chemokines implicated in leukocyte recruitment and maintenance within the inflamed skin. Consistent with these transcriptional changes, immunoblotting of lesional skin lysates showed that DNCB increased inflammasome‐associated proteins, including NLRP3, caspase‐1, IL‐1β, and IL‐18 (Figure 5D). Dexa reduced the DNCB‐associated induction of these proteins. TR treatment also attenuated this DNCB‐associated increase in inflammasome‐associated proteins, with a clearer reduction observed for NLRP3 and IL‐1β relative to that of the DNCB group. In contrast, caspase‐1 and IL‐18 exhibited comparatively modest and/or inconsistent changes across TR doses in this dataset. As cleaved or mature active forms were not distinguished, these data indicate reduced inflammasome‐associated protein induction rather than definitive suppression of inflammasome activation. Collectively, these data suggest that TR reduces lesional chemokine transcript expression and attenuates inflammasome‐associated protein induction in DNCB‐challenged skin.

### 3.6. TR Reduces MEK/ERK and JAK2–STAT1 Phosphorylation in DNCB‐Challenged Tissue

**Figure 6:**
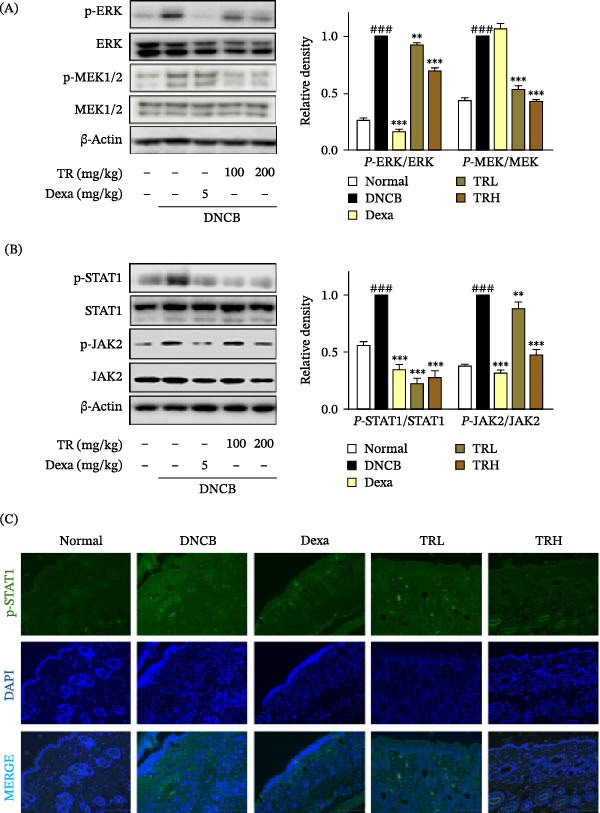
Effects of TR on MEK/ERK and JAK2–STAT1 phosphorylation and p‐STAT1 immunoreactivity in DNCB‐challenged mice. (A) Immunoblotting of MEK/ERK phosphorylation status and corresponding total proteins in dorsal skin. (B) Immunoblotting of JAK2–STAT1 phosphorylation status and corresponding total proteins. β‐actin served as an internal control. (C) Immunofluorescent detection of p‐STAT1 in skin sections with DAPI counterstaining. Quantification of band intensity was conducted by densitometry and expressed as normalized relative density. Data are presented as the mean ± SD. Densitometric analysis was conducted using ImageJ software. The results are expressed as the mean ± SD. ###*p* < 0.001 versus the normal control group;  ^∗∗^ *p* < 0.01 and  ^∗∗∗^ *p* < 0.001 versus the DNCB‐treated group.

Figure 6 summarizes the pathway‐focused validation of DNCB‐induced signaling. DNCB challenge increased the phosphorylation of MEK1/2 and ERK, consistent with activation of the MAPK cascade in lesional skin (Figure 6A). Both Dexa and TR reduced the phosphorylation of these targets relative to the DNCB group. Across the TR doses, the higher‐dose regimen showed a more consistent decrease in phospho‐signals, while the corresponding total proteins exhibited comparatively limited variation, indicating that the principal observed change was on phosphorylation status. DNCB also increased the phosphorylation of JAK2 and STAT1 (Figure 6B). Dexa substantially suppressed these activation markers. TR decreased p‐JAK2 and p‐STAT1 compared with DNCB, with a dose‐dependent pattern favoring 200 mg/kg. Total JAK2 and STAT1 remained largely preserved when normalized to β‐actin, supporting an association with reduced phosphorylation rather than a global suppression of total protein abundance. Immunofluorescence staining corroborated the biochemical readouts: the p‐STAT1 signal was accentuated in DNCB‐challenged tissue and diminished in the Dexa and TR groups (Figure 6C). Collectively, these data indicate that TR is associated with reduced MEK/ERK and JAK2–STAT1 activation in DNCB‐induced dermatitis, in a manner that is more apparent at the higher dose. However, without pathway intervention experiments, these data indicate pathway‐associated changes rather than causal pathway dependence.

### 3.7. TR Dampens TNF‐α/IFN‐γ–Induced Inflammatory Signaling and Modulates AhR Protein Abundance in HaCaT Keratinocytes

**Figure 7:**
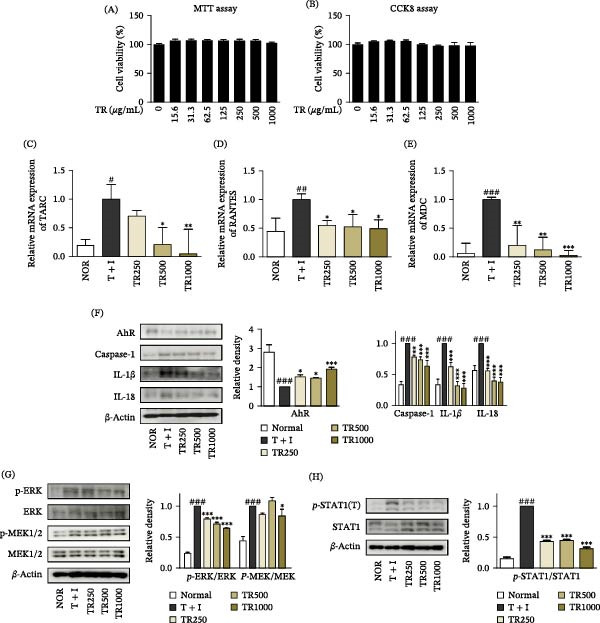
Effects of TR on cytokine‐driven inflammatory programs in TNF‐α/IFN‐γ‐treated HaCaT keratinocytes. (A, B) Cell viability of HaCaT keratinocytes treated with TR at 0–1000 μg/mL, determined using MTT (A) and CCK‐8 (B) assays. Viability is expressed as a percentage of the untreated control. (C–E) Relative mRNA expression of TARC/CCL17 (C), RANTES/CCL5 (D), and MDC/CCL22 (E) in TNF‐α/IFN‐γ‐stimulated HaCaT keratinocytes treated with TR. Gene expression levels were normalized to GAPDH and expressed relative to the TNF‐α/IFN‐γ‐stimulated group. (F) Representative immunoblots and densitometric quantification of AhR and inflammasome‐associated proteins, including caspase‐1, IL‐1β, and IL‐18. (G) Representative immunoblots and densitometric quantification of MEK/ERK signaling proteins, including p‐MEK1/2, total MEK1/2, p‐ERK, and total ERK. (H) Representative immunoblots and densitometric quantification of STAT1 signaling proteins, including p‐STAT1 (Tyr701) and total STAT1. For (F–H), densitometric quantification is summarized as relative density, with total proteins normalized to β‐actin and phosphorylated targets normalized to their corresponding total proteins, as applicable. Data are presented as the mean ± SD from three independent experiments; densitometric analysis was conducted using ImageJ software. For (C–H), #*p* < 0.05, ##*p* < 0.01, and ###*p* < 0.001 versus the normal control group;  ^∗^ *p* < 0.05,  ^∗∗^ *p* < 0.01, and  ^∗∗∗^ *p* < 0.001 versus the TNF‐α/IFN‐γ‐stimulated group.

Cell viability was assessed using both MTT and CCK‐8 assays under the same exposure conditions as those used for subsequent signaling experiments. TR did not significantly reduce HaCaT cell viability at 250, 500, or 1000 μg/mL (Figure 7A,B). Therefore, these concentrations were used to evaluate cytokine‐induced inflammatory signaling in TNF‐α/IFN‐γ‐stimulated HaCaT keratinocytes. Consistent with the lesional skin qRT‐PCR findings, TNF‐α/IFN‐γ stimulation markedly increased the mRNA expression of AD‐associated chemokines, including TARC/CCL17, MDC/CCL22, and RANTES/CCL5, in HaCaT keratinocytes. TR treatment significantly reduced the cytokine‐induced expression of these chemokine transcripts, supporting the ability of TR to attenuate keratinocyte‐derived inflammatory chemokine responses at the transcriptional level (Figure 7C–E). At the protein level, TNF‐α/IFN‐γ stimulation increased key inflammatory effectors, including caspase‐1, IL‐1β, and IL‐18 (Figure 7F). TR reduced the intensity of these bands compared with TNF‐α/IFN‐γ stimulation alone, indicating the attenuation of the cytokine‐induced inflammatory protein program. In the same samples, AhR signal decreased with TNF‐α/IFN‐γ stimulation and increased with TR cotreatment, consistent with partial reversal of cytokine‐driven AhR suppression. Pathway readouts further aligned with these changes. TNF‐α/IFN‐γ stimulation elevated the phosphorylation of MEK1/2 and ERK (Figure 7G) and markedly increased p‐STAT1 (Tyr701) (Figure 7H). TR reduced phosphorylation across these signaling nodes with limited changes in total protein abundance, indicating that TR acts predominantly on signal activation rather than global protein expression. Overall, the combined viability, immunoblot, and phosphorylation data support that TR dampens TNF‐α/IFN‐γ triggered signaling and inflammasome‐related protein induction in HaCaT keratinocytes. Because canonical AhR target genes, AhR transcriptional activity, and pathway intervention experiments were not assessed, these findings should be interpreted as pathway‐associated changes rather than evidence of direct AhR activation or causal pathway dependence.

## 4. Discussion

By integrating target‐space overlap analysis with in vivo and in vitro validation, this study supports the hypothesis that TR extract may have multitarget modulatory potential in AD‐relevant immunometabolic circuitry rather than acting as a single‐pathway inhibitor. Common‐target filtering identified 25 genes shared between the predicted TR target space and the AD gene space, and their PPI topology formed a compact multiedge module enriched for transcriptional regulators and signal‐transduction mediators (e.g., PPARG, PPARD, NR1H3, MAPK3, PTPN6, PTPN11, and NOS2), suggesting, at the prediction level, that TR could influence coordinated signaling–transcription programs in AD. In enrichment analyses, immune activation terms (including T cell receptor and IFN‐γ–linked processes) coappeared with lipid metabolic and nuclear receptor–related terms, suggesting that the overlap set may not be confined to a single immune axis but may instead map to pathways coupling immune outputs with metabolic transcriptional control. This pattern is consistent with established evidence that barrier‐associated lipid programs and inflammatory signaling are coupled in AD pathophysiology [6], and it may provide a conceptual bridge between the observed enrichment profile and the barrier readouts assessed in vivo. Nevertheless, these network pharmacology results should be interpreted as prediction‐based and hypothesis‐generating, whereas the experimental findings in this study are based on the observed anti‐inflammatory and barrier‐related effects of TR in the DNCB‐induced AD‐like mouse model and TNF‐α/IFN‐γ‐stimulated HaCaT keratinocytes.

At the organismal level, repeated DNCB challenge produced a robust dermatitis‐like phenotype, and TR treatment reduced lesion severity in serial photography and lowered clinical dermatitis scores during the treatment phase. Endpoint TEWL was measured as an objective indicator of epidermal permeability barrier function as TEWL is widely used to assess skin barrier function in inflammatory dermatoses, including AD [19]. Notably, Dexa improved gross inflammatory features but remained associated with elevated TEWL in this dataset, indicating that gross anti‐inflammatory improvement did not fully coincide with TEWL normalization under the present experimental conditions. Although previous experimental studies have reported that glucocorticoids may affect epidermal lipid synthesis and permeability barrier recovery [20], the present study did not directly assess lipid synthesis or barrier recovery kinetics. Therefore, these TEWL findings should be interpreted as endpoint‐level evidence of barrier function alteration rather than as a comprehensive assessment of permeability barrier recovery dynamics. Histologically, DNCB induced marked epidermal hyperplasia and increased dermal mast cell numbers, whereas TR decreased epidermal thickness and reduced mast cell abundance relative to DNCB with a dose‐responsive trend. Because epidermal hyperplasia and inflammatory cell accumulation are common readouts across sensitizer‐driven dermatitis models, the directionality of these histologic improvements supports a tissue‐level anti‐inflammatory effect of TR that is not restricted to subjective scoring [21].

The chemokine results further strengthen this interpretation, as DNCB elevated the lesional mRNA expression of TARC/CCL17, MDC/CCL22, and RANTES/CCL5, and TR suppressed TARC and RANTES dose‐dependently, with a clearer reduction of MDC at the higher dose. These specific chemokines are clinically and mechanistically relevant, given that serum TARC levels correlate with AD disease activity (SCORAD) and decrease with effective treatment, and TARC is detected in keratinocytes and dermal immune/stromal compartments in AD [22]. Similarly, serum MDC/CCL22 is elevated in AD, correlates with SCORAD and other inflammatory indices, including soluble E‐selectin and soluble IL‐2 receptor, and decreases after treatment, supporting MDC as a disease‐activity–linked Th2‐associated chemokine in humans [23]. RANTES/CCL5 is increased in lesional atopic eczema skin along with its receptors CCR3 and CCR5, and its expression is linked to eosinophil infiltration in challenged and unchallenged lesions, indicating that the RANTES axis participates in chronic inflammatory cell recruitment in atopic skin [24]. Accordingly, the TR‐associated reduction in TARC/CCL17, MDC/CCL22, and RANTES/CCL5 transcript expression in lesional skin is consistent with attenuation of a transcriptional chemokine response associated with the recruitment and retention of CCR4‐ and CCR3/CCR5‐associated leukocyte subsets, although the present study did not phenotype infiltrating T cells or eosinophils directly [22–24]. Consistent with these tissue findings, TR also reduced TNF‐α/IFN‐γ‐induced TARC/CCL17, MDC/CCL22, and RANTES/CCL5 mRNA expressions in HaCaT keratinocytes. However, because the corresponding chemokine protein levels were not measured in lesional skin tissues, these findings should be interpreted as transcript‐level evidence of chemokine modulation. Beyond chemokine tone, TR also blunted a DNCB‐driven inflammasome‐associated protein signature in the skin, reducing the abundance of NLRP3, caspase‐1, IL‐1β, and IL‐18 relative to the DNCB group. This finding is mechanistically relevant because keratinocytes can participate in NLRP3‐associated inflammatory responses to cutaneous stressors, including contact sensitizers, thereby contributing to epithelial amplification of skin inflammation [25, 26]. In AD‐like inflammation models, attenuation of the NLRP3 inflammasome axis has been associated with reduced dermatitis phenotypes and decreased IL‐1β/IL‐18‐related inflammatory readouts, supporting the relevance of this axis beyond contact‐sensitizer settings [27]. Moreover, emerging evidence suggests that NLRP3 may also contribute to AD‐like inflammation through noncanonical mechanisms, such as transcriptional regulation of IL‐33 in keratinocytes, indicating that NLRP3‐associated biology in the skin may extend beyond canonical inflammasome assembly alone [28]. Therefore, the coordinated reduction of NLRP3 and inflammasome‐associated cytokine proteins by TR suggests the attenuation of an epithelial inflammatory protein signature. However, because activation‐specific readouts were not assessed, these findings should be interpreted as reduced inflammasome‐associated protein expression rather than definitive suppression of inflammasome activation.

Consistent with the target‐space prediction that MAPK signaling is a central intersection point (MAPK3/ERK1 emerging as a hub), TR reduced DNCB‐induced phosphorylation of MEK1/2 and ERK while leaving the total protein abundance relatively stable, indicating preferential modulation of pathway activation rather than global protein suppression. The biological significance of ERK modulation in AD is supported by evidence that phosphorylated ERK is highly expressed in mouse and human AD skin and that topical ERK pathway inhibition can improve dermatitis severity, TEWL, and filaggrin expression in an AD‐like mouse model [29]. In parallel, TR reduced the phosphorylation of JAK2 and STAT1 in lesional skin and decreased p‐STAT1 immunofluorescence, aligning the experimental data with the network‐level enrichment of IFN‐γ–linked processes. IFN‐γ expression in atopic eczema lesions has been documented at the mRNA level and shown to decline with clinical improvement, supporting the relevance of IFN‐γ–responsive transcriptional programs in at least a subset of AD disease states [30]. At the cellular level, IFN‐γ activates JAK2–STAT1 signaling in keratinocytes and can induce antigen presentation–related programs, providing a mechanistic framework in which dampening JAK2–STAT1 phosphorylation could reduce inflammatory epithelial outputs [31]. The presence of PTPN6 (SHP‐1) and PTPN11 (SHP‐2) among the central overlap nodes provides an additional layer of coherence because SHP‐1 is a negative regulator of IL‐4/IL‐13–activated JAK–STAT signaling and SHP‐2 promotes Ras–MAPK pathway activation downstream of diverse receptors [32, 33]. Although the current experiments do not establish direct target engagement, the alignment between phosphatase‐centric network hubs and observed decreases in ERK and STAT phosphorylation strengthens the biological plausibility for TR‐associated modulation of proximal signaling control points [33]. The nuclear receptor hubs in the overlap set (PPARD and NR1H3/LXRα) also contextualize the lipid‐related enrichment and provide a bridge to barrier outcomes because topical activation of PPARβ/δ and LXR has been shown to reverse clinical dermatosis, improve TEWL and stratum corneum hydration, and normalize multiple immunologic abnormalities in an oxazolone‐induced AD‐like dermatitis model [34]. Furthermore, PPARγ activation can stimulate keratinocyte differentiation, increase epidermal differentiation markers, including filaggrin, and accelerate barrier recovery following acute disruption in mice, supporting the broader premise that nuclear receptor signaling can couple barrier repair with anti‐inflammatory effects [35]. However, the observation that PPARγ activators were not effective in the oxazolone AD‐like model highlights that the functional contribution of individual PPAR isoforms is context‐dependent, and it cautions against assuming that hub status in a network necessarily implies a straightforward therapeutic directionality [34]. Mechanistically, LXRα activation has been reported to induce lipogenesis in HaCaT keratinocytes, providing a plausible cellular‐level link between NR1H3‐associated signaling and epidermal lipid production that could influence barrier phenotypes such as TEWL [36]. The KEGG enrichment for efferocytosis is additionally intriguing because efferocytosis is a central component of inflammation resolution biology, and macrophage efferocytosis is associated with the upregulation of PPAR and LXR signaling components [37, 38]. While efferocytosis was not directly assessed here, the co‐occurrence of efferocytosis and nuclear receptor pathways in the overlap space offers a testable hypothesis that TR may not only suppress inflammatory initiation but could also bias the tissue milieu toward pro‐resolving programs [37]. The HaCaT experiments provide direct support for epithelial‐intrinsic effects of TR under inflammatory stimulation, as TR was well tolerated at the concentrations used for signaling analyses and attenuated TNF‐α/IFN‐γ–induced increases in caspase‐1, IL‐1β, and IL‐18 while reducing phosphorylation of MEK/ERK and STAT1 with limited changes in total protein levels. This in vitro paradigm is relevant because TNF‐α/IFN‐γ stimulation of HaCaT cells is known to induce chemokines, including TARC/CCL17, MDC/CCL22, and RANTES/CCL5, and to activate STAT1 and ERK signaling [39, 40], thereby modeling a keratinocyte inflammatory state that overlaps with pathways highlighted in the network analysis. Accordingly, the TR‐associated reduction of STAT1 and ERK phosphorylation in keratinocytes complements the in vivo phospho‐signaling results and supports the inference that TR may modulate keratinocyte signal transduction‐associated responses, not solely through systemic immunosuppression [39, 40]. TR also restored AhR protein abundance that was reduced under TNF‐α/IFN‐γ stimulation, but the present data do not establish whether TR activates AhR transcriptional programs or whether AhR changes are secondary to reduced inflammatory signaling. This distinction matters because AhR activation in keratinocytes from AD patients has been reported to normalize filaggrin expression via OVOL1, providing a mechanistic rationale to interrogate whether TR‐associated AhR changes translate into barrier gene regulation [41]. Future mechanistic work should therefore couple AhR protein measurements to canonical AhR target gene readouts and to barrier‐differentiation outputs to avoid over‐attribution of causality [41].

From a chemical standardization perspective, L‐citrulline was used in this study as a marker compound for HPLC–ELSD‐based quality control of the TR water extract rather than as evidence that L‐citrulline is the sole or principal active anti‐AD constituent. L‐citrulline was selected because amino acids, including L‐citrulline, have been reported as characteristic constituents of *Trichosanthes kirilowii* and have been used in the analytical characterization of TR‐related materials [13, 42]. In contrast, trichosanthin is a previously reported protein component from *Trichosanthes* species with immunomodulatory activity, but it was not directly detected or quantified in the present hot‐water extract. Moreover, its stability and content under the present extraction conditions were not examined. Therefore, the biological effects observed in this study should be interpreted as the effects of the TR water extract as a whole, while the specific active constituents responsible for the anti‐AD activity remain to be identified through future fractionation‐guided and constituent‐based studies.

From a pharmacognosy perspective, prior reports on TR‐derived constituents support the plausibility that a multicomponent TR preparation can modulate both redox‐ and immune‐linked pathways [14–18]. Our findings extend this rationale by directly testing TR water extract in AD‐like dermatitis models and linking in vivo barrier‐associated/inflammation readouts to ERK‐, JAK–STAT‐, and NLRP3‐associated signaling changes. Nevertheless, extract composition, bioavailability, and target engagement should be defined to support reproducibility and translational interpretation [14]. Several limitations should be considered when interpreting the current findings, beginning with the fact that network pharmacology and target‐space overlap analyses rely on predicted or literature‐derived interactions that can be incomplete or biased toward well‐studied proteins. Second, the DNCB‐induced dermatitis model captures key features of eczematous inflammation and barrier disruption but is a sensitizer‐driven model and does not fully recapitulate the clinical heterogeneity of human AD, necessitating validation in complementary AD‐like models [21, 43]. Third, although chemokine suppression and pathway phosphorylation readouts support immunomodulatory activity, the study did not quantify canonical type‐2 cytokines or detailed immune cell phenotypes, which limits inference about which immune circuits are most directly affected by TR [21]. Fourth, TEWL improvement suggests barrier‐associated functional benefit, but the study did not directly measure stratum corneum lipid composition or differentiation markers, which would be required to connect nuclear receptor–linked pathway enrichment to a defined barrier repair mechanism [34]. Fifth, the TR preparation was evaluated as an extract, and without chemical standardization and constituent quantification, it remains unclear which molecules drive the observed effects and whether batch‐to‐batch variation could alter efficacy or safety. Addressing these limitations will require multimodel validation in AD‐relevant systems, incorporation of lipidomic and transcriptomic endpoints, and mechanistic perturbation studies to determine whether the implicated ERK, JAK–STAT, and NLRP3‐associated pathways are causally required for the observed effects of TR using pharmacologic inhibitors or genetic approaches [27, 29]. In parallel, defining extract chemistry and testing purified fractions or constituents will be essential to establish reproducible exposure–response relationships and to support future development of TR‐derived topical candidates as barrier‐sparing anti‐inflammatory adjuncts [14].

## 5. Conclusions

This study supports TR as a potential multicomponent intervention for AD‐like skin inflammation. TR attenuated clinical dermatitis severity, scratching behavior, epidermal hyperplasia, mast cell accumulation, lesional chemokine transcript expression, and inflammasome‐associated protein induction in DNCB‐challenged mice while reducing cytokine‐induced chemokine responses and inflammatory signaling‐related proteins in HaCaT keratinocytes. HPLC–ELSD‐based marker analysis further supported the quality control characterization of the TR water extract using L‐citrulline as a marker compound. These findings suggest that TR may mitigate AD‐like inflammation in association with epithelial inflammatory, chemokine‐related, and barrier‐associated functional responses. As an extract‐level pharmacological study, the present work does not assign these effects to L‐citrulline, trichosanthin, a single purified constituent, or a definitive pathway‐specific mechanism. Further fractionation‐guided standardization and target validation studies will help define the active constituents responsible for the pharmacological effects and the molecular basis of TR activity.

## Author Contributions

**Hye-Min Kim**: methodology, investigation, data curation, formal analysis, visualization, writing – original draft, writing – review and editing, funding acquisition. **Geun-Lip Kim**: conceptualization, investigation, data curation. **Tae-Young Gil**: conceptualization, methodology, investigation, validation. **Ha-Yeon Sim**: data curation, formal analysis, visualization. **Hwan Lee**: investigation, data curation, visualization. **Dong-Sung Lee**: methodology, supervision, validation. **Kyou-Young Lee**: supervision, validation. **Hyo-Jin An**: conceptualization, methodology, validation, supervision, project administration.

## Funding

This research was supported by a grant of the Korea Health Technology R&D Project through the National Research Foundation (NRF), the Korea Health Industry Development Institute (KHIDI), funded by the Ministry of Science and ICT, the Republic of Korea, and the Ministry of Health and Welfare, Republic of Korea (Grant RS‐2023‐00262645).

## Disclosure

All data were generated in‐house, and no paper mill was used. All authors agree to be accountable for all aspects of the work, ensuring integrity and accuracy.

## Ethics Statement

This study was approved by the Institutional Animal Care and Use Committee (IACUC) of Kyung Hee University (Approval Number KHMC‐IACUC‐2023‐027). All procedures were conducted in accordance with applicable national regulations and the ARRIVE guidelines. Animals were housed under standard conditions, and all efforts were made to minimize pain, distress, and the number of animals used. Appropriate anesthesia and euthanasia methods were employed.

## Conflicts of Interest

The authors declare no conflicts of interest.

## Supporting Information

Additional supporting information can be found online in the Supporting Information section.

## Supporting information


**Supporting Information** Supporting Information Table S1: TR compounds identified in the TCMSP database. Supporting Information Table S2: Predicted target genes of the TR ingredients in the SwissTargetPrediction database. Supporting Information Table S3: AD‐related target genes in the GeneCards database. Supporting Information Table S4: Overlapping genes of TR and AD. Supporting Information Table S5: Centrality scores of the top 10 hub genes identified by cytoHubba. Supporting Information Table S6: GO functional enrichment of TR in the DAVID database. Supporting Information Table S7: KEGG pathways of TR in the DAVID database. Supporting Information Table S8: List of primer sequences for mouse and human.

## Data Availability

The data that support the findings of this study are available from the corresponding author upon reasonable request.
